# Conceptus elongation in ruminants: roles of progesterone, prostaglandin, interferon tau and cortisol

**DOI:** 10.1186/2049-1891-5-53

**Published:** 2014-11-16

**Authors:** Kelsey Brooks, Greg Burns, Thomas E Spencer

**Affiliations:** Department of Animal Science and Center for Reproductive Biology, Washington State University, Pullman, WA 99164 USA

**Keywords:** Conceptus, Cortisol, Endometrium, Interferon, Prostaglandin, Ruminant

## Abstract

The majority of pregnancy loss in ruminants occurs during the first three weeks after conception, particularly during the period of conceptus elongation that occurs prior to pregnancy recognition and implantation. This review integrates established and new information on the biological role of ovarian progesterone (P4), prostaglandins (PGs), interferon tau (IFNT) and cortisol in endometrial function and conceptus elongation. Progesterone is secreted by the ovarian corpus luteum (CL) and is the unequivocal hormone of pregnancy. Prostaglandins (PGs) and cortisol are produced by both the epithelial cells of the endometrium and the trophectoderm of the elongating conceptus. In contrast, IFNT is produced solely by the conceptus trophectoderm and is the maternal recognition of pregnancy signal that inhibits production of luteolytic pulses of PGF_2α_ by the endometrium to maintain the CL and thus production of P4. Available results in sheep support the idea that the individual, interactive, and coordinated actions of P4, PGs, IFNT and cortisol regulate conceptus elongation and implantation by controlling expression of genes in the endometrium and/or trophectoderm. An increased knowledge of conceptus-endometrial interactions during early pregnancy in ruminants is necessary to understand and elucidate the causes of infertility and recurrent early pregnancy loss and provide new strategies to improve fertility and thus reproductive efficiency.

## Introduction

This review integrates established and new information on the biological role of ovarian progesterone (P4), prostaglandins (PGs), interferon tau (IFNT) and cortisol in endometrial function and conceptus elongation during the peri-implantation period of pregnancy in ruminants. Our knowledge of the complex biological and genetic mechanisms governing conceptus elongation and implantation remains limited in domestic ruminants [1], but is essential to ameliorate early pregnancy losses and increase fertility of ruminants.

Establishment of pregnancy in domestic ruminants (i.e., sheep, cattle, goats) begins at the conceptus stage and includes pregnancy recognition signaling, implantation, and placentation [2–5]. The morula-stage embryo enters the uterus on days 4 to 6 post-mating and then forms a blastocyst that contains an inner cell mass and a blastocoele or central cavity surrounded by a monolayer of trophectoderm. After hatching from the zona pellucida (days 8 to 10), the blastocyst slowly grows into a tubular or ovoid form and is then termed a conceptus (embryo-fetus and associated extraembryonic membranes) [5, 6]. In sheep, the ovoid conceptus of about 1 mm in length on day 11 begins to elongate on day 12 and forms a filamentous conceptus of 15 to 19 cm or more in length by day 15 that occupies the entire length of the uterine horn ipsilateral to the corpus luteum (CL) with extraembryonic membranes extending into the contralateral uterine horn. In cattle, the hatched blastocyst forms an ovoid conceptus between days 12 to 14 and is only about 2 mm in length on day 13. By day 14, the conceptus is about 6 mm, and the elongating bovine conceptus reaches a length of about 60 mm (6 cm) by day 16 and is 20 cm or more by day 19. Thus, the bovine blastocyst/conceptus doubles in length every day between days 9 and 16 with a significant increase (~30-fold) in length between days 12 and 15 [7, 8]. In both sheep and cattle, conceptus elongation involves exponential increases in length and weight of the trophectoderm [9] and onset of extraembryonic membrane differentiation, including gastrulation of the embryo and formation of the yolk sac and allantois that are vital for embryonic survival and formation of a functional placenta [5, 6]. Trophoblast elongation observed in ruminants appears to not involve geometrical changes in cell shape but rather occurs from cell proliferation [10]. Successively, the elongated conceptus begins the process of central implantation and placentation around day 16 in sheep and day 19 in cattle [11].

Blastocyst growth into an elongated conceptus does not occur *in vitro*, as it requires secretions supplied by the endometrium of the uterus [12–14]. The uterine luminal fluid (ULF) contains substances, collectively termed histotroph, that govern elongation of the conceptus via effects on trophectoderm proliferation and migration as well as attachment and adhesion to the endometrial luminal epithelium (LE) [4, 15, 16]. Histotroph is derived primarily from transport and (or) synthesis and secretion of substances by the endometrial LE and glandular epithelia (GE), and it is a complex and rather undefined mixture of proteins, lipids, amino acids, sugars (glucose, fructose), ions and exosomes/microvesicles [17–21]. The recurrent early pregnancy loss observed in uterine gland knockout (UGKO) ewes established the importance of uterine epithelial-derived histotroph for support of conceptus elongation and implantation [14]. Available evidence supports the idea that ovarian P4 induces expression of a number of genes, specifically in the endometrial epithelia, that are then further stimulated by factors from the conceptus (e.g., IFNT, PGs, cortisol) as well as the endometrium itself (e.g., PGs and cortisol) [22]. The genes and functions regulated by these hormones and factors in the endometrial epithelia elicit specific changes in the intrauterine histotrophic milieu necessary for conceptus elongation [4, 15, 16, 22, 23].

### Progesterone regulation of endometrial function and conceptus elongation

Progesterone stimulates and maintains endometrial functions necessary for conceptus growth, implantation, placentation, and development to term. In cattle, concentrations of P4 during early pregnancy clearly affect embryonic survival [13, 24]. In both lactating dairy cows and heifers, there is a strong positive association between the post-ovulatory rise in P4 and embryonic development. Increasing concentrations of P4 after ovulation enhanced conceptus elongation in beef heifers [25, 26], dairy cows [27], and sheep [28], while lower P4 concentrations in the early luteal phase retarded embryonic development in sheep and cattle [24, 29, 30]. Supplementation of cattle with P4 during early pregnancy has equivocal effects to increase embryonic survival [31] and is unlikely to rescue development of embryos with inherent genetic defects or in high-producing dairy cows [27, 32, 33].

Progesterone predominantly exerts an indirect effect on the conceptus via the endometrium to regulate blastocyst growth and conceptus elongation [28, 30, 34–36]. Similar to humans [37, 38], the endometria of both cyclic and pregnant sheep and cattle express genes implicated in uterine receptivity, which can be defined as a physiological state of the uterus when conceptus growth and implantation for establishment of pregnancy is possible. The absence of a sufficiently developed conceptus to signal pregnancy recognition results in those genes being turned ‘off’ as luteolysis ensues and the animal returns to estrus for another opportunity to mate. The outcome of the P4-induced changes in the cyclic and pregnant uterus is to modify the intrauterine milieu, such as an increase in select amino acids, glucose, cytokines and growth factors in histotroph, for support of blastocyst growth into an ovoid conceptus and elongation to form a filamentous conceptus [4, 15, 22, 23].

#### Sheep

**Figure 1:**
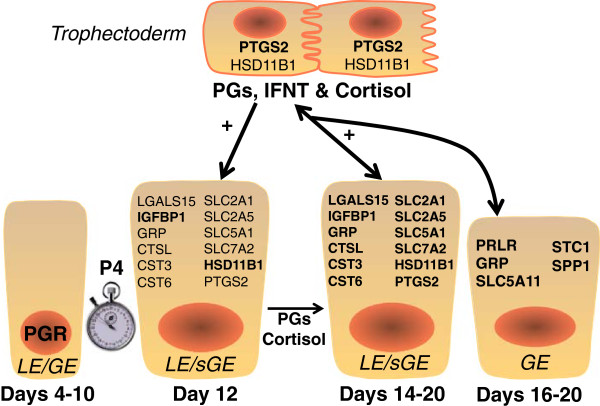
**Schematic illustrating the effects of ovarian hormones and factors from the endometrium and conceptus trophectoderm on expression of elongation- and implantation-related genes in the endometrial epithelia of the ovine uterus during early pregnancy.** Progesterone action for 8–10 days down-regulate expression of the progesterone receptor (PGR). The loss of PGR is correlated with the induction of many genes in the endometrial LE and sGE, including PTGS2 and HSD11B1 involved in prostaglandin (PG) and cortisol production, respectively, in both cyclic and pregnant ewes. If the ewe is pregnant, the trophectoderm synthesizes and secretes PGs, interferon tau (IFNT), and cortisol that act on the endometrium in a cell-specific manner to up-regulate the expression of many P4-induced genes that govern endometrial functions and/or elongation of the conceptus. Legend: GE, glandular epithelia; IFNT, interferon tau; LE, luminal epithelium; PG, prostaglandins; PGR, progesterone receptor; sGE, superficial glandular epithelia.

**Figure 2:**
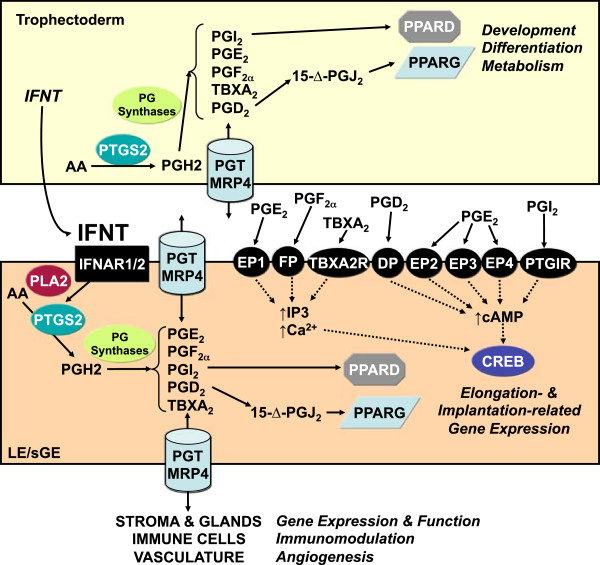
**Schematic illustrating working hypothesis of the biological role of interferon tau (IFNT) and prostaglandins (PGs) in uterine function and conceptus elongation during early pregnancy in sheep.** See text for detailed description. Legend: ABCC4, ATP-binding cassette, sub-family C (CFTR/MRP), member 4; CREB, cAMP responsive element binding protein; IFNAR, interferon (alpha, beta and omega) receptor; DP, prostaglandin D receptor (PTGDR); EP, prostaglandin E receptor (PTGER); FP, prostaglandin F receptor (PTGFR); IP, prostaglandin I receptor (PTGIR); PLA2, phospholipase A2; PPARD, peroxisome proliferator-activated receptor delta; PPARG, peroxisome proliferator-activated receptor gamma; PTGS2, prostaglandin-endoperoxide synthase 2 (prostaglandin G/H synthase and cyclooxygenase); PG Synthases, prostaglandin synthases (AKR1C3, PTGDS, PTGES, PTGFS, PTGIS, TBXAS); SLCO2A1, solute carrier organic anion transporter family, member 2A1 (prostaglandin transporter); TBXA2R, thromboxane A2 receptor.

Actions of ovarian P4 on the uterus are essential for conceptus survival and growth in sheep [28]. Between days 10 and 12 after onset of estrus or mating in cyclic and pregnant ewes, P4 induces the expression of many conceptus elongation- and implantation-related genes (Figure 1 and Table 1). The initiation of expression of those genes requires P4 action and is temporally associated with the loss of progesterone receptors (PGR) between days 10 and 12 in the endometrial LE and between days 12 and 14 to 16 in the GE after onset of estrus; however, PGR remain present in the stroma and myometrium in the ovine uterus throughout pregnancy [39]. In the endometrial LE and superficial GE (sGE), P4 induces genes that encode secreted attachment and migration factors (galectin-15 [LGALS15] and insulin-like growth factor binding protein one [IGFBP1]), intracellular enzymes (prostaglandin G/H synthase and cyclooxygenase 2 [PTGS2] and hydroxysteroid (11-beta) dehydrogenase 1 [HSD11B1]), secreted proteases (cathepsin L1 [CTSL1]), secreted protease inhibitors (cystatin C [CST] 3 and 6), a secreted candidate cell proliferation factor (gastrin releasing peptide [GRP]), glucose transporters (SLC2A1, SLC2A5, SLC5A1), and a cationic amino acid (arginine, lysine and ornithine) transporter (SLC7A2) [3, 4, 15]. In the endometrial GE, P4 induces genes that encode for a secreted cell proliferation factor (GRP), a glucose transporter (SLC5A11), secreted adhesion protein (secreted phosphoprotein one or SPP1), a candidate regulator of calcium/phosphate homeostasis (stanniocalcin one or STC1), and a potential immunomodulatory factor (SERPINA14, also known as uterine milk proteins or uterine serpins) [3, 4, 15]. Several of those P4-induced genes in the epithelia are further stimulated by the actions of PGs, IFNT and/or cortisol, resulting in changes in components of the uterine luminal fluid histroph that regulate conceptus elongation via effects on trophectoderm proliferation and migration (Figures 1 and 2).

**Table 1:** **Effects of ovarian progesterone (P4) and intrauterine infusion of interferon tau (IFNT), prostaglandins (PGs) or cortisol on elongation- and implantation-related genes expressed in the endometrial epithelia of the ovine uterus** ^**1**^

Gene symbol	P4	IFNT	PGs ^2^	Cortisol
Transport of glucose				
*SLC2A1*	↑	+	+	+
*SLC2A5*	n.d.	n.e.	+	+
*SLC2A12*	n.d.	+	n.e. or +	+
*SLC5A1*	↑	+	n.e. or +	+
*SLC5A11*	↑	+	n.e. or +	n.e.
Transport of amino acids				
*SLC1A5*	n.d.	n.d.	+	+
*SLC7A2*	↑	+	n.e.	n.e.
Cell proliferation, migration and (or) attachment				
*GRP*	↑	+	+	+
*IGFBP1*	↑	+	++	n.e.
*LGALS15*	↑	++	++	++
*SPP1*	↑	+	n.d.	++
Proteases and their inhibitors				
*CTSL1*	↑	++	n.e. or +	+
*CST3*	↑	+	n.e. or +	n.e.
*CST6*		+	n.e.	+
Enzymes				
*HSD11B1*	↑	+	++	+
*PTGS2*	↑	n.e. (+ activity)	n.e. (+ activity)	n.e. (+ activity)

^1^Effect of hormone or factor denoted as induction (↑), stimulation (+ or ++), no effect (n.e.), decrease (**-**) or not determined (n.d.). ^2^Summary data for infusion of PGE2, PGF2α, or PGI2 [40].

#### Cattle

Comparisons of the endometrial transcriptome in cyclic and pregnant heifers (days 5, 7, 12 and 13) found no difference prior to pregnancy recognition (days 15 or 16) [41, 42]. Indeed, the major changes required to drive conceptus elongation and establish uterine receptivity to implantation occur between days 7 and 13 in response to ovarian P4, irrespective of whether an appropriately developed embryo/conceptus is present or not [23, 30, 41, 43–46]. Similar to sheep, PGR protein is lost from the LE by day 13 and in the GE by day 16, and PGR loss is associated with the down- and up-regulation of genes expressed in the endometrial epithelia [47]. Using a global gene profiling approach, studies have identified the temporal changes that occur in endometrial gene expression in both cyclic [30] and pregnant [43] heifers following an elevation or diminution of post-ovulatory P4 during metestrus that promotes or delays conceptus elongation, respectively [30, 34, 48]. As summarized in a recent review [23], the expression of several genes is lost in the LE and GE, including PGR and a protease (alanyl (membrane) aminopeptidase [ANPEP]), and in the GE, a lipase (lipoprotein lipase [LPL]), protease (matrix metallopeptidase 2 [MMP2]) and immunomodulatory protein with antimicrobial activity (lactotransferrin [LTF]), between days 7 and 13 after onset of estrus or mating in cyclic and pregnant heifers. As expected, many conceptus elongation- and implantation-related genes appear in the endometrial epithelia between days 7 and 13 in cyclic and pregnant heifers. Genes up-regulated in the LE encode a mitogen (connective tissue growth factor [CTGF]) and in the GE encode a transport protein (retinol binding protein 4 [RBP4]), a glucose transporter (SLC5A1), and a protein involved in transport and cell proliferation (fatty acid binding protein 3 [FABP3]). Further, some genes are up regulated in both the LE and GE that encode secreted attachment and migration factors (lectin, galactoside-binding, soluble, 9 [LGALS9] and insulin-like growth factor binding protein one [IGFBP1]) as well as an intracellular enzyme (PTGS2). Those gene expression changes in the endometrium elicit changes in the ULF histotroph that are hypothesized to support conceptus elongation [23, 49, 50]. It is quite clear that substantial differences in gene expression occur between the receptive endometrium of sheep and cattle, as one of the most abundant genes (LGALS15) induced by P4 and stimulated by IFNT in the endometrium of sheep is not expressed in cattle [51]. However, PTGS2 and IGFBP1 are common uterine receptivity markers and regulators of conceptus elongation in both sheep and cattle [46, 52]. Of note, *in vitro* produced bovine embryos will elongate when transferred into a receptive ovine uterus [53].

### Interferon tau regulation of endometrial function and conceptus elongation

Maternal recognition of pregnancy is the physiological process whereby the conceptus signals its presence to the maternal system and prolongs the lifespan of the ovarian CL [54]. In ruminants, IFNT is the pregnancy recognition signal secreted by the elongating conceptus that acts on the endometrium to inhibit development of the luteolytic mechanism [15, 16, 55, 56]. Interferon tau is secreted predominantly by the elongating conceptus before implantation [57, 58]. The antiluteolytic effects of IFNT inhibit transcription of the *estrogen receptor alpha* (*ESR1*) gene in sheep and *oxytocin receptor* (*OXTR*) gene in both sheep and cattle specifically in the endometrial LE. The absence of OXTR in the endometrium prevents the release of luteolytic pulses of PGF2α, thereby ensuring maintenance of the CL and continued production of P4 [3, 59]. Although IFNT inhibits *OXTR* expression, it does not inhibit expression of *PTGS2*, which is important for the generation of PGs that are critical regulators of conceptus elongation during early pregnancy [60]. In addition to antiluteolytic effects, IFNT acts in a paracrine manner on the endometrium to induce or enhance expression of IFN-stimulated genes (ISGs) that are hypothesized to regulate uterine receptivity and conceptus elongation and implantation [4, 40, 61, 62].

#### Classical type I IFN-stimulated genes in the endometrium

A number of transcriptional profiling experiments conducted with human cells, ovine endometrium, bovine endometrium, and bovine peripheral blood lymphocytes have elucidated classical ISG induced by IFNT during pregnancy [3, 4, 41, 42, 63]. In cattle, comparisons of days 15 to 18 pregnant and non-pregnant or cyclic endometria revealed conceptus effects on endometrial gene expression, particularly the induction or up-regulation of classical ISGs [23, 41–43, 64, 65]. In sheep, *ISG15* (ISG15 ubiquitin-like modifier) is expressed in LE of the ovine uterus on days 10 or 11 of the estrous cycle and pregnancy, but is not detected in the LE by days 12 to 13 of pregnancy [66]. In response to IFNT from the elongating conceptus, *ISG15* is induced in the stratum compactum stroma and GE by days 13 to 14, and expression extends to the stratum spongiosum stroma, deep glands, and myometrium as well as resident immune cells of the ovine uterus by days 15 to 16 of pregnancy [66, 67]. As IFNT production by the conceptus trophectoderm declines, expression of ISG in the stroma and GE also declines, but some remain abundant in endometrial stroma and GE on days 18 to 20 of pregnancy. Similar temporal and spatial alterations in *ISG15* expression occur in the bovine uterus during early pregnancy [68, 69].

*In vivo* studies revealed that the majority of classical ISG (*B2M*, *GBP2, IFI27, IFIT1, ISG15, IRF9, MIC, OAS*, *RSAD2*, *STAT1,* and *STAT2*) are not induced or up-regulated by IFNT in endometrial LE or sGE of the ovine uterus during early pregnancy [66, 70–73]. This finding was initially surprising, because all endometrial cell types express *IFNAR1* (interferon [alpha, beta and omega] receptor 1) and *IFNAR2* subunits of the common Type I IFN receptor [74]. Further, bovine endometrial, ovine endometrial, and human 2fTGH fibroblast cells were used to determine that IFNT activates the canonical janus kinase-signal transducer and activator of transcription-interferon regulatory factor (JAK-STAT-IRF) signaling pathway used by other Type I IFNs [75]. About the same time, it was discovered that IRF2, a potent transcriptional repressor of ISGs [76], is expressed specifically in the endometrial LE and sGE and represses transcriptional activity of genes containing IFN-stimulated response element (ISRE)-containing promoters [70, 77]. In fact, all components of the ISGF3 transcription factor complex (*STAT1, STAT2, IRF9*) and other classical ISGs (*B2M, GBP2, IFI27, IFIT1, ISG15, MIC, OAS*) contain one or more ISRE in their promoters. Thus, IRF2 in LE appears to restrict IFNT induction of most classical ISG to stroma and GE of the ovine uterus. The silencing of *MIC* and *B2M* genes in endometrial LE or sGE during pregnancy may be a critical mechanism preventing immune rejection of the semi-allogeneic conceptus [71]. As IRF2 is not expressed in other uterine cell types, classical ISGs are substantially increased in the endometrial stroma, GE and immune cells by IFNT from the conceptus during early pregnancy. Of particular note, several reports indicate induction or increases in ISGs in peripheral blood lymphocytes and the CL during pregnancy of sheep and cattle or in ewes receiving intrauterine injections of IFNT [61, 63]. Recent evidence indicates that IFNT exits the uterus to exert systemic effects that alter maternal physiology, including function of the CL [61, 78–80].

One challenge has been to determine which of the large number of classical ISGs induced in the endometrium by IFNT have a biological role in conceptus-endometrial interactions, as traditionally the main function of Type I IFN is to inhibit viral infection and has primarily been associated with cellular antiviral responses [81]. One classical ISG with reported biological effects on trophectoderm growth and adhesion in ruminants is *CXCL10* [chemokine (C-X-C motif) ligand 10; alias IP-10], a member of the C-X-C chemokine family that regulates multiple aspects of inflammatory and immune responses primarily through chemotactic activity toward subsets of leukocytes [82, 83]. ISG15 conjugates to intracellular proteins through a ubiquitin-like mechanism [40], and deletion of *Isg15* in mice results in 50% pregnancy loss manifest during early placentation [84]. In addition, MX proteins are thought to regulate secretion through an unconventional secretory pathway [85]. The enzymes which comprise the 2′,5′-oligoadenylate synthetase (OAS) family regulate ribonuclease L antiviral responses and may play additional roles in control of cellular growth and differentiation [72].

#### Non-classical IFNT-stimulated genes in the endometrium

Although IFNT is the only known IFN to act as the pregnancy recognition signal, IFNs appear to have a biological role in uterine receptivity, decidualization, and placental growth and development in primates, ruminants, pigs, and rodents [40, 62]. Transcriptional profiling of human U3A (STAT1 null) cells and ovine endometrium, as well as candidate gene analyses were used to discover novel ‘non-classical’ ISG in the endometrial LE during pregnancy such as *CST3, CTSL*, *HSD11B1, IGFBP1, LGALS15* and *WNT7A* (wingless-type MMTV integration site family, member 7A) [28, 86–90]. Subsequently, a series of transcriptomic and candidate gene studies found that IFNT stimulates expression of a number of elongation- and implantation-related genes that are initially induced by P4 (*CST3, CST6, CTSL, GRP, HSD11B1, IGFBP1, LGALS15, SLC2A1, SLC2A5, SLC5A11, SLC7A2*) specifically in the endometrial LE, sGE, and (or) GE [3, 4, 62, 90] (Figure 1). None of these genes are classical Type I ISG, and are referred to as ‘non-classical or novel’ ISG. Indeed, IFNT stimulation of these non-classical ISG requires induction by P4 and loss of PGR in the epithelia. Importantly, all of the non-classical ISG encode factors that have actions on the trophectoderm (proliferation, migration, attachment and (or) adhesion, nutrient transport) important for conceptus elongation (Table 1). For example, knockdown of an arginine transporter (SLC7A1) in the conceptus trophectoderm and inhibition of PTGS2 or HSD11B1 activity in utero compromised conceptus elongation in sheep [60, 91, 92]. The effects of IFNT in the bovine endometrium are not as well understood in terms of non-classical ISGs, but recent studies have started to unravel those effects in cattle [41, 42, 93].

Given that the critical signaling components of the JAK-STAT signaling system (STAT1, STAT2, IRF9) are not expressed in endometrial LE or sGE [70], IFNT must utilize a noncanonical, STAT1-independent signaling pathway to regulate expression of genes in endometrial LE and sGE of the ovine uterus. The noncanonical pathway mediating IFNT stimulation of genes in the endometrial LE and sGE has not been entirely elucidated, but other Type I IFN utilize mitogen-activated protein kinase (MAPK) and phosphatidylinositol 3-kinase (PI3K) cascades [94]. Available evidence suggests that IFNT activates distinct epithelial and stromal cell-specific JAK, epidermal growth factor receptor, MAPK (ERK1/2), PI3K-AKT, and (or) Jun N-terminal kinase (JNK) signaling modules to regulate expression of PGE_2_ receptors in the endometrium of the ovine uterus or in ovine uterine LE cells *in vitro*[95, 96]. As discussed subsequently, recent evidence indicates that PTGS2-derived PGs and HSD11B1-derived cortisol are part of the noncanonical pathway of IFNT action on the endometrium in sheep [60, 97].

### Prostaglandin regulation of endometrial function and conceptus elongation

Results of recent studies in sheep support the concept that PGs regulate expression of elongation- and implantation-related genes in the endometrial epithelia of ruminants during early pregnancy and are involved in conceptus elongation [46, 60, 98] (Figures 1 and 2). The conceptus and endometria synthesize a variety of PGs during early pregnancy in both sheep and cattle [99–104]. The endometrium produces and uterine lumen contains substantially more PGs during early pregnancy than during the estrous cycle [105–107]. The dominant cyclooxygenase expressed in both the endometrium and trophectoderm of the elongating conceptus is PTGS2 [104–106]. Although the antiluteolytic effects of IFNT are to inhibit expression of the *OXTR* in the endometrial LE and sGE of early pregnant ewes, it does not impede up-regulation of PTGS2, a rate-limiting enzyme in PG synthesis [102, 107]. In sheep, PTGS2 activity in the endometrium is stimulated by IFNT, and PTGS2-derived PG were found to mediate, in part, the effects of P4 and IFNT on the endometrium of the ovine uterus. In those studies, the abundance of *HSD11B1* and *IGFBP1* mRNA in the endometrium was considerably reduced by intrauterine infusion of meloxicam, a selective PTGS2 inhibitor. As illustrated in Figure 1, *PTGS2* expression appears between days 10 and 12 post-estrus and mating in the endometrial LE and sGE and is induced by ovarian P4 [98, 102]. In the bovine uterus, PTGS2 is also not down-regulated in endometria of early pregnant cattle, but rather is up-regulated by IFNT [108, 109]. Thus, IFNT acts as a molecular switch that stimulates PGE_2_ production in the bovine endometrium [110]. Indeed, Type I IFNs were found to stimulate phospholipase A2 (PLA_2_) and synthesis of PGE_2_ and PGF_2α_ in several different cell types over 25 years ago [111, 112].

Prostaglandins are essential for conceptus elongation, as intrauterine infusions of meloxicam prevented conceptus elongation in early pregnant sheep [60, 98]. The elongating conceptuses of both sheep and cattle synthesize and secrete more PG than the underlying endometrium [99, 100, 113]. Thus, PG levels are much greater in the uterine lumen of pregnant as compared with cyclic or nonpregnant cattle [106]. In sheep, Charpigny and coworkers [103] found that PTGS2 was abundant in day 8 to 17 blastocysts/conceptuses, whereas PTGS1 was undetectable. PTGS2 protein increased in the conceptus trophectoderm between days 8 and 14 and was maximal between days 14 and 16. In fact, there was a 30-fold increase in PTGS2 content per protein extract between days 10 and 14, corresponding to a 50,000-fold increase in the whole conceptus, and PTGS2 protein in the conceptus then declined substantially after Day 16 to become undetectable by day 25 of pregnancy. Other studies found that Day 14 sheep conceptuses *in vitro* release mainly cyclooxygenase metabolites including PGF2α, 6-keto-PGF1α (i.e., a stable metabolite of PGI_2_), and PGE_2_[103], and day 16 conceptuses produce substantially more of those PGs than day 14 conceptuses [101]. Given that membrane and nuclear receptors for PGs are present in all cell types of the ovine endometrium and conceptus during early pregnancy [60, 114], PTGS2-derived PGs from the conceptus likely have paracrine, autocrine, and perhaps intracrine effects on endometrial function and conceptus development during early pregnancy (Figure 2). Indeed, expression of *PTGS2* in biopsies of day 7 bovine blastocysts is a predictor of the successful development of that blastocyst to term and delivery of a live calf [114]. Further, pregnancy rates were substantially reduced in heifers that received meloxicam, a partially selective inhibitor of PTGS2, on day 15 after insemination [115]. Thus, PGs are critical regulators of conceptus elongation and implantation in ruminants, as they are for blastocyst implantation and decidualization during pregnancy in mice, rats, hamsters, mink and likely humans [116–118].

Recently, Dorniak and coworkers [52] infused PGE_2_, PGF_2α_, PGI_2_, or IFNT, at the levels produced by the day 14 conceptus, into the uterus of cyclic ewes. In that study, expression of *GRP*, *IGFBP1*, and *LGALS15* were increased by PGE_2_, PGI_2_, and IFNT, but only IFNT increased *CST6* (Table 1). Differential effects of PG were also observed for *CTSL1* and its inhibitor *CST3*. For glucose transporters, IFNT and all PG increased *SLC2A1*, but only PG increased *SLC2A5* expression, whereas *SLC2A12* and *SLC5A1* were increased by IFNT, PGE_2_, and PGF_2α_. Infusions of all PGs and IFNT increased the amino acid transporter *SLC1A5*, but only IFNT increased *SLC7A2*. In the uterine lumen, only IFNT increased glucose levels, and only PGE_2_ and PGF_2α_ increased total amino acids [52]. Thus, available results support the idea that PG and IFNT from the conceptus coordinately regulate endometrial functions important for growth and development of the conceptus during the peri-implantation period of pregnancy [22] (Figures 1 and 2).

Prostaglandins also have intracrine effects within cells. Both PGI_2_ and PGJ_2_ can activate nuclear peroxisome proliferator-activating receptors (PPARs) [119]. PGI_2_ is a ligand for PPARD, and PGD_2_ spontaneously forms 15-deoxy-Δ12,14-PGJ_2_ within cells that is a ligand for PPARG [120–123]. PPARs dimerize with retinoid X receptors (RXRs) and regulate transcription of target genes. Although PGs are lipid-derived, their efflux out and influx into cells depends on specific PG transporters (PGT) termed solute carrier organic anion transporter family, member 20A1 (SLC20A1) and ATP-binding cassette, sub-family C (CFTR/MRP), member 4 (ABCC4 or MRP4). PGJ_2_ and PGI_2_ are not as efficiently transported as other PGs (PGE_2_, PGF_2α_, TBXA_2_). Expression of prostacyclin (PGI2) synthase (PTGIS), PGI2 receptors (PTGIR), PPARs and RXRs in uteri and conceptuses of sheep during early pregnancy has been well documented [124]. In the endometrium, *PTGIS* mRNA and protein were expressed mainly in the endometrial LE/sGE as early as day 9 of pregnancy, but levels declined from days 12 to 17. Expression of PTGIR, PPARs (PPARA, PPARD, PPARG) and RXRs (RXRA, RXRB, RXRG) was detected in the endometrium, and PPARD and PPARG were particularly abundant in the endometrial LE and sGE. In the conceptus trophectoderm, *PTGIS* expression increased and then peaked at day 17. *PTGIR* and *PPARA* mRNAs peaked before day 12 and then declined and were nearly undetectable by Day 17, whereas *PPARD* and *PPARG* mRNAs increased from Days 12 to 17 in the conceptus. These results suggest that PPARG may also regulate conceptus trophectoderm development and differentiation due to intrinsic actions of PGJ_2_, which is spontaneously formed within cells from PGD_2_.

Unexpectedly, genetic studies in mice found that *Pparg* is essential for placental development, as null mutation of *Pparg* in mice resulted in placentae with poor differentiation and vascular anomalies, leading to embryonic death by gestational day 10 [123]. In mink, treatment of trophoblast cells with PGJ_2_ attenuated cell proliferation, increased PPARG expression, elicited the appearance of enlarged and multinuclear cells, and increased the expression of adipophilin or ADRP (adipose differentiation-related protein), a protein involved in lipid homeostasis, and SPP1 [125]. PPARs alter the transport, cellular uptake, storage, and use of lipids and their derivatives [119]. In extravillous cytotrophoblasts of human placentae, PPARG stimulates synthesis of chorionic gonadotrophin (hCG) and increases free fatty acid (FFA) uptake. PPARG-regulated genes include fatty acid binding proteins (FABP) and fatty acid transport proteins [FATP or SLC27As] required for lipid uptake and triacylglycerol synthesis, which is undoubtedly important in rapidly growing and elongating conceptuses producing large amounts of PGs.

Mice deficient in *Ppard* also exhibit placental defects and reduced or inhibited trophoblast giant cell differentiation [126, 127]. PPARD is activated by PGI_2_, and treatment of rat trophoblast cells with a specific PPARD agonist triggered early differentiation of giant cells that expressed CSH1 (chorionic somatomammotropin hormone one or placental lactogen) and reduced expression of inhibitor of differentiation two (ID2), which is an inhibitor of several basic helix-loop-helix (bHLH) transcription factors, such as HAND1, that promote giant cell differentiation. Further, PPARD increases expression of ADRP, and PPARD potentiates cell polarization and migration in the skin [128], which are all cellular activities implicated in conceptus elongation. Thus, PTGS2-derived PGs and PPARG may impact conceptus elongation via effects on trophectoderm growth and survival as well as expression of elongation- and implantation-related genes in the endometrial epithelia.

### Cortisol regulation of endometrial function and conceptus elongation

Initially identified as a candidate P4-regulated gene in the endometrium that potentially governed conceptus elongation [36, 52], *HSD11B1* was found to be expressed specifically in the endometrial LE and sGE and is induced by P4 and stimulated by IFNT and PGs in the endometrium of the ovine uterus [98] (Table 1). Expression of *HSD11B1* is also up-regulated in the endometrium of cattle between days 7 and 13 of pregnancy [43]. One of two isoforms of hydroxysteroid (11-beta) dehydrogenases that regulate intracellular levels of bioactive glucocorticoids within key target tissues [129], HSD11B1 is a low affinity NADP(H)-dependent bidirectional dehydrogenase/reductase for glucocorticoids, and the direction of HSD11B1 activity is determined by the relative abundance of NADP^+^ and NADPH co-factors. The endometrium of the ovine uterus as well as conceptus generates active cortisol from inactive cortisone [97]. Cortisol regulates gene expression via the nuclear receptor subfamily 3, group C, member 1 (NR3C1 or glucocorticoid receptor [GR]), a transcriptional regulator that modulates expression of primary target genes that either directly affect cellular physiology or alter the expression of other secondary target genes, which then confer hormonal responses [130, 131].

Recent findings support the idea that PGs mediate, in part, P4 induction and IFNT stimulation of *HSD11B1* expression in the ovine endometrium [60, 97]. Similarly, PG regulate activity of HSD11B1 in bovine endometria [132], and PGF_2α_ stimulates the activity of HSD11B1 in human fetal membranes [133, 134]. Whereas PG stimulate HSD11B1 activity, glucocorticoids enhance PG synthesis by up-regulating expression and activity of PLA_2_ and PTGS2 in the ovine placenta, thereby establishing a positive feed-forward loop implicated in the timing of parturition [135]. This tissue-specific stimulatory role of glucocorticoids on PG synthesis contradicts the classical concept that glucocorticoids exert anti-inflammatory effects on immune cells [136].

Available results support the idea that cortisol from the endometrium as well as conceptus regulates endometrial functions important for conceptus elongation during early pregnancy in sheep. The day 14 conceptus expresses both *HSD11B1* and *HSD11B2* as well as *NR3C1*[60, 98]. Indeed, the elongating sheep conceptus generates cortisol from cortisone via HSD11B1, and elevated levels of cortisol are found in the uterine lumen of early pregnant sheep [97]. Thus, cortisol may have paracrine and intracrine effects on the endometrium and conceptus trophectoderm during early pregnancy (Figure 1). As summarized in Table 1, intrauterine infusions of cortisol at early pregnancy levels into the uterus of cyclic ewes from day 10 to 14 post-estrus increased the expression of several elongation- and implantation-related genes expressed in the endometrial epithelia of the ovine uterus and increased endometrial PTGS2 and HSD11B1 expression and/or activity [92]. Similar to IFNT actions, PTGS2-derived PGs mediated some effects of cortisol. In order to determine if HSD11B1-derived cortisol is important for conceptus elongation, PF915275, a selective HSD11B1 inhibitor, was infused into the uterine lumen of bred ewes from days 8 to 14 post-mating [92]. Inhibition of HSD11B1 activity in utero prevented conceptus elongation. Thus, HSD11B1-derived cortisol is an essential regulator of conceptus elongation via effects on trophectoderm growth and survival as well as expression of elongation- and implantation-related genes in the endometrial epithelia.

The effect of knocking out NR3C1 in the elongating conceptus has not been reported in ruminants. Indeed, NR3C1 targets hundreds of genes, including those involved in lipid metabolism and triglyceride homeostasis, in other organs and cell types [130, 137]. In humans, the proposed positive roles of HSD11B1-generated cortisol at the conceptus-maternal interface include stimulation of hormone secretion by the trophoblast, promotion of trophoblast growth/invasion, and stimulation of placental transport of glucose, lactate and amino acids. Indeed, glucocorticoids can have positive as well as negative effects during pregnancy [138]. Administration of synthetic glucocorticoids to women during pregnancy can alter normal development of the fetus and compromise pregnancy success by inhibiting cytokine-PG signaling, restricting trophoblast invasion, and inducing apoptosis in placenta. Similarly, administration of synthetic glucocorticoids to pregnant ewes reduced placental growth and development, numbers of trophoblast giant binucleate cells in the placenta, and circulating levels of placental lactogen [139]. On the other hand, natural glucocorticoids are hypothesized to have positive effects during early pregnancy [138]. Interestingly, administration of glucocorticoids increased pregnancy rates in women undergoing assisted reproductive technologies and pregnancy outcomes in women with a history of recurrent miscarriage [140, 141].

## Conclusions

The individual, additive and synergistic actions of P4, IFNT, PGs and cortisol regulate expression of elongation- and implantation-related genes in the endometrial epithelia in ruminants. The outcome of the carefully orchestrated changes in gene expression is secretion or transport of substances (e.g., glucose, amino acids, proteins) from the endometrium into the uterine lumen that govern conceptus survival and elongation via effects on trophectoderm proliferation, migration, attachment, and adhesion. Moreover, conceptus elongation is also likely governed by intracrine factors and pathways such as PGs and PPARs. A systems biology approach is necessary to fully understand conceptus elongation and the multifactorial phenomenon of early pregnancy loss. Such information is critical to provide a basis for new strategies to improve the fertility and reproductive efficiency in ruminant livestock.

### Ethical approval

This is a review paper; however, all results reported based on research by the authors was approved by the Washington State University Institutional Animal Care and Use Committee.
